# GRK2 activity promotes Aβ generation by altering GPR3 signaling and intracellular trafficking

**DOI:** 10.1002/alz.095165

**Published:** 2025-01-09

**Authors:** William Lu, Thais Rafael Guimaraes, Yunhong Huang, Nicholas Todd, Amantha Thathiah

**Affiliations:** ^1^ University of Pittsburgh, Pittsburgh, PA USA

## Abstract

**Background:**

G protein‐coupled receptors (GPCRs) are associated with multiple stages of the pathophysiology of Alzheimer’s disease (AD). Biased GPCR signaling preferentially activates G protein‐ or β‐arrestin‐mediated signaling pathways and presents opportunities to develop more selective and safer therapeutics but remains largely unexplored in AD. Recently, we developed a G protein‐biased GPR3 AD mouse model, which does not recruit β‐arrestin 2, that displays reduced amyloid‐β (Aβ) pathology without adverse cognitive effects associated with elimination of both G protein and β‐arrestin signaling.

**Method:**

We investigated how G protein‐biased GPR3 signaling ameliorates Aβ accumulation in HEK293 cells stably expressing the full‐length amyloid precursor protein (HEK‐APP) using biochemical and immunocytochemical methods. To investigate the involvement of GPCR kinases (GRKs) in GPR3‐ mediated Aβ generation, we employed a CRISPR/Cas9 genome editing strategy to generate *Grk2*‐deficient HEK293 cells along with complementary pharmacological inhibition of GRK2 in HEK‐APP and human neurons.

**Result:**

We first validated that biased GPR3 signaling reduces Aβ generation in HEK‐APP cells. To gain mechanistic insight into how biased GPR3 affects Aβ generation, we determined that biased GPR3 displays reduced binding to APP relative to wild‐type GPR3. We then determined that *Grk2* deficiency leads to reduced β‐arrestin 2 recruitment to GPR3 and decreased Aβ generation in HEK‐APP cells. Similar to the effect of Grk2 deficiency, pharmacological inhibition of GRK2 activity with CMPD101 also leads to a reduction in Aβ generation in both HEK‐APP cells and familial AD neurons. Further studies indicate that GRK2 modulates the localization of GPR3 and APP to the Golgi apparatus, early endosomes and endoplasmic reticulum. Interestingly, GRK2 inhibition also leads to increased levels of APP phosphorylation at threonine 668.

**Conclusion:**

These results suggest a role for GRK2 in regulating the intracellular trafficking and localization of G protein‐biased GPR3 and APP, which may directly affect Aβ generation. Future studies will further elucidate how GRK2 activity impacts GPR3‐mediated Aβ generation and could illuminate novel targets for the development of AD therapies.

